# Functional gastrointestinal disorder is associated with increased non-gastrointestinal healthcare consumption in the general population

**DOI:** 10.1111/j.1742-1241.2007.01549.x

**Published:** 2008-02

**Authors:** T Ålander, K Svärdsudd, L Agréus

**Affiliations:** 1 Center for Family and Community Medicine, Department of Neurobiology, Caring Sciences and Society, Karolinska InstituteHuddinge, Sweden; 2 Ture Ålander Family PracticeUppsala, Sweden; 3 Department of Public Health and Caring Sciences, Unit of Family Medicine, University HospitalUppsala, Sweden

## Abstract

**Objectives:**

Comparison of comorbidity and healthcare consumption in primary healthcare subjects with persistent functional gastrointestinal disorder (FGID) and a strictly gastrointestinal (GI) symptom-free group (SSF).

**Methods:**

A stratified sample (*n* = 1428, 21–86 years) of subjects living in the Östhammar community, Sweden, was limited to half of the community and classified through the Abdominal Symptom Questionnaire (ASQ) into two study groups, one with persistent FGID (*n* = 71), another SSF (*n* = 48). Symptoms were re-evaluated by means of the ASQ at a surgery visit, as was healthcare consumption during 2 years, and the levels of anxiety and depression, as measured with the Hospital Anxiety and Depression Scale. Diagnoses were set according to The International Classification of Diseases (ICD)-9 and the 14 diagnostic groups.

**Results:**

Of the FGID patients, 97% had a non-GI diagnosis, compared with 100% of SSF (ns). The mean number of doctors’ consultations (OR = 3.5), phone calls to doctors (OR = 3.4), number of prescriptions (OR = 2.4) and number of set diagnoses (OR = 3.9), anxiety level (OR = 11.5) and depression (OR = 5.2) were all statistically significantly higher (p < 0.05) for FGID than for SSF, while the number of referrals and sick leave were not. Besides a GI diagnosis, there was no significant difference (p > 0.05) in the spectrum of morbidity in terms of ICD-9 subgroup classification, except an increased proportion of older SSF subjects with circulatory disorders and hypertension.

**Conclusions:**

Functional gastrointestinal disorders are related to an increased demand on primary healthcare because of an increased overall comorbidity, which signifies a need for a holistic healthcare approach.

**What's known**

Previous studies have shown an association between FGID and other ‘medically unexplained physical symptoms’ such as migraine, fibromyalgia, pelvic pain etc. There is also an increased healthcare seeking reported for people with FGID.

**What's new**

This article is, to the authors’ knowledge, that the first report which compares primary healthcare consumption in persons with persistent FGID with strictly GI symptom-free controls. The findings indicate the importance of looking for other physical morbidity in patients who present themselves with FGID.

## Introduction

Gastrointestinal (GI) symptoms are common, and in Sweden and other Western countries (1–3) have an estimated prevalence of 50% for dyspepsia, irritable bowel syndrome (IBS) and gastroesophageal reflux disease (GERD) together. Many sufferers report more than one of the disorders concomitantly (4). The disorders, although often intermittent, are costly for the society (5,6) and lingering for many of the sufferers (3,7,8). However, the absence of GI complaints appear stable over time (3,8): a symptom survey conducted in Sweden over 7 years determined that from subjects who were symptom free at the beginning only 3% developed functional gastrointestinal disorders (FGID) and 3% reflux disease after 1 year, and this increased to 5% in each group after 7 years (8). Community surveys state that less than half of GERD or dyspepsia sufferers ever consult (1,3), whereas, those with IBS appear to initiate consultation more frequently (3). Together, FGID account for one of 20 visits in primary care (1,9,10).

Functional dyspepsia is defined as dyspepsia without peptic ulcer disease (PUD) or other more rare diseases (11). In a Swedish population-based upper endoscopy study, the prevalence of dyspepsia was 38% and symptomatic PUD was 4% (12). Thus, a majority of those reporting dyspepsia in the population can be expected to have functional dyspepsia. Similarly, the symptoms of IBS in most cases represent a FGID (13). Moreover, dyspepsia and IBS often overlap in individuals (4); thus, dyspepsia and IBS probably have common aspects in their pathophysiology (14,15) and also in healthcare seeking behaviour (16). By contrast, GERD, in the majority of cases, has an acid-induced aetiology (13). Accordingly, it appears reasonable to investigate the burden imposed on society by functional dyspepsia and IBS together, excluding those with only GERD.

Psychological and other non-GI somatic illnesses occur in FGID sufferers more frequently; conversely, GI disorders are associated with other diseases such as fibromyalgia, headache, gynaecological disorders (17) and psychological illness (18). As many FGID sufferers seldom or never consult about their GI symptoms, but rather for other complaints (19), their entire medical record data must be examined to understand all aspects of their comorbidity.

The aim of this study was to compare the comorbidity of subjects with persistent FGID with those of a strictly GI symptom-free group (SSF), and to compare their healthcare consumption, as registered in their primary care medical records. The FGID and SSF study groups were identified with the Abdominal Symptom Questionnaire (ASQ) from a random sample of Östhammar population in 1995 and the study groups were further evaluated with new questionnaires at a visit to their local health centre.

## Materials and methods

### Setting and sampling

Östhammar community (*n* = 22,452 in 1995) is served by five primary healthcare centres, three to the west (Gimo, Österbybruk and Alunda) and two to the east (Öregrund and Östhammar city, *n* = 9959 in 1995); the latter two serve almost half of the population. The two eastern health centres were the only centres included in this study, as they were in a more remote area serving the inhabitants with 24 h primary care, including emergency care. Moreover, the medical records were computerised, making data collection more reliable. The eastern part did not differ from the western population in terms of age (mean age: east 49 years, west 48 years and p = 0.86) or gender (males: east 39%, west 39% and p = 1). However, the mean education level was higher (east 3.1, west 2.7 and p = 0.01), although the median = 3 was the same.

From a questionnaire survey of a random sample (*n* = 1428, 21–86 years, mean age 49.2 years, 47% males) of Östhammar community in 1995, and repeated in 1996, a total of 141 FGID and 97 SSF were identified for further study: 71 FGID and 48 SSF lived in the eastern part of the community. Details of the sampling procedure and study logistics have been reported previously (18). Those with FGID (mean age 45 years, range: 21–85 years) were younger than the SSF (mean age 54 years, range: 24–82 years and p < 0.001), but the gender distribution was equal (males: 39% FGID, 40% SSF and p = 0.99).

### Symptom group definitions

The symptom profile of each person was classified through self-reported troublesome symptoms during the previous 3 months entered in the postal ASQ (20). Dyspepsia was defined as ≥ 1 of 11 listed pain and discomfort modalities (burning sensation, aching, pain, tenderness, sinking feeling, ‘butterflies’, cramp, twinge, stitch, colic or gripes) at or above the navel level, and concomitantly reporting ≥ 1 of the symptoms: acid reflux, heartburn, retrosternal pain, eructations, nausea, vomiting, early satiety, uncomfortable feeling of fullness after meals or abdominal distension. Subjects reporting only gastroesophageal reflux symptoms (heartburn and/or retrosternal pain) but no concomitant abdominal pain or discomfort were classified as having GERD and not dyspepsia: those with such symptoms and concomitant abdominal pain or discomfort were classified into the dyspepsia group.

Irritable bowel syndrome was defined as having ≥ 1 of the 11 pain and discomfort modalities listed above and in any abdominal location. In addition, ≥ 1 of the symptoms, diarrhoea, constipation or alternating diarrhoea and constipation, and ≥ 1 of the symptoms abdominal distension, abdominal discomfort or pain on defaecation, abdominal discomfort or pain relieved by defaecation, feeling of incomplete defaecation or mucous stools. This definition is in concordance with published guidelines at the time of the survey (21).

### Functional gastrointestinal disorder

Functional gastrointestinal disorders was defined as having dyspepsia, IBS or both. The occurrence of IBS and dyspepsia within the FGID group (*n* = 71) was: in 1995; IBS *n* = 2; dyspepsia *n* = 43; IBS and dyspepsia *n* = 26 and in 1996; IBS *n* = 8; dyspepsia *n* = 25; IBS and dyspepsia *n* = 38.

### Strictly symptom free

Strictly symptom free was defined as having no reported symptom in the ASQ in the 1995 survey, and having stated that they had had no previous troubling abdominal symptoms. Those subjects who had participated in two former surveys in 1988 and 1989 should also have reported no symptoms in both of those two investigations.

### Data collection

Treatment diagnoses and the number of contacts were extracted from medical records between 1st January 1996 and 31st December 1997.

### Coding diagnosis

The ICD-9 code was categorised into 14 disease diagnostic groups. As ICD-9 coding was not compulsory, a code could be absent in the medical records and was interpreted from the text. If a person had a diagnosis code from the same diagnostic group on repeated consultations, the diagnostic group was only registered once.

### Counting contacts

Doctor phone calls, face-to-face consultations, consultation diagnoses, prescriptions, referrals, sick listing periods and sick listing days were obtained from the medical records.

### Anxiety and depression

Anxiety and depression were measured by a validated questionnaire: Hospital Anxiety and Depression Scale (HADS) (22) with possible ranges of 0–21 for each subscale. There is no single, generally accepted reference score for HADS; the cut-off is dependent on the sensitivity and specificity adopted (23). In this study, the lower cut-off 7/8 for anxiety and 6/7 for depression were chosen.

### Statistical power and analysis

To have 90% power at the p < 0.05 level to detect a 100% absolute difference in mean consultation rate, 72 subjects in the FGID and 36 subjects in the SSF groups were needed. This assumed an annual consultation rate of two for the FGID and one for the SSF group, a SD = 3 in both groups and twice as many subjects in the FGID group than in the SSF group. Pearson's chi-squared test, Student's *t*-test, Fisher's exact test and the Mann–Whitney *U*-test (for data with a skewed distribution) were used for the statistical analyses. Logistic regression was analysed with age, sex, education, depression and anxiety as independent variables and healthcare factors (doctor consultations, phone calls, prescriptions, referrals, sick leave episodes and number of different diagnosis) as dependent variables: all variables dichotomised. Ninety-five per cent confidence intervals (CI) were computed with parametric methods: a p-value of 0.05 or less was considered statistically significant and all reported p-values were two sided. The statistical package Stata 8 was used for analyses (24).

## Ethics

This study was part of the GiCon study approved by the Ethics Committee of the Medical Faculty, Uppsala University, on 5th June 1996.

## Results

### Doctor face-to-face and phone consultations

For any disorder there were 300 consultations for the FGID entered in the medical records and 110 consultations for the SSF groups. The healthcare actions are summarised in Table 1.

**Table 1:** Healthcare actions per person for functional gastrointestinal disorder (FGID) and strictly symptom free (SSF) 1996–1997 and reported mood disorder. Odds ratios for FGID compared with SSF (=1) adjusted for age, sex, education, anxiety and depression: healthcare actions are dependent variables, all introduced into a multivariate analysis with the logistic regression method

	Doctor consultations* (*n* = 410)	Phone calls* (*n* = 103)	Prescription* (*n* = 552)	Referrals* (*n* = 34)	Sick leave episodes* (*n* = 56)	Sick leave days* (*n* = 1204)	Diagnoses* (*n* = 247)	Anxiety†	Depression†
FGID (*n* = 71)	4.2 (3.2–5.3)	1.1 (0.8–1.5)	6.0 (4.3–7.8)	0.3 (0.2–0.5)	0.6 (0.1–1.1)	13.2 (0–31)	2.5 (2.0–3.0)	6.2 (6) (5.2–6.3)	3.9 (3); (2.9–4.0)
SSF (*n* = 48)	2.3 (1.5–3.1)	0.5 (0.2–0.8)	3.7 (1.8–5.6)	0.2 (0.1–0.4)	0.3 (0–0.7)	5.5 (0–14)	1.5 (1.0–2.0)	3.2 (3) (2.9–3.5)	1.5 (1); (1.3–2.1)
p (Mann–Whitney)	0.004	0.004	0.02	0.4	0.2	0.5	0.002	< 0.0001	0.0001
FGID (SSF = 1)‡	3.5 (0.007);(1.4–8.6)	3.4 (0.008);(1.4–8.3)	2.4 (0.036);(1.1–5.5)	1.6 (0.30);(0.6–4.2)	1.6 (0.69);(0.4–5.7)	0.71 (0.70); (0.1–4.1)	2.9 (0.022);(1.2–7.3)	11.5 (0.025);(1.4–96)	5.2 (0.038);(1.1–25)

* Values given are mean (95% CI).

† Values given are mean (median); (95% CI).

‡ Values given are OR (p) (95% CI).

Those with FGID consulted a doctor more often, made more phone calls, received more diagnoses and obtained more prescriptions than the SSF group. The distribution of consultations and phone calls is presented in Table 2, and prescriptions and diagnoses in Table 3.

**Table 2:** Number of consultations and phone calls for subjects with functional gastrointestinal disorder (FGID) and strictly symptom free (SSF) 1996–1997

Number of doctor consultations and phone calls	FGID consultation (*n* = 300)	%	SSF consultation (*n* = 110)	%	FGID calls (*n* = 81)	%	SSF calls (*n* = 22)	%
11–20	8	11	1	2	–	–	–	–
7–10	5	7.0	4	8	–	–	–	–
3–6	24	34	10	21	15	21	4	8
1–2	23	32	15	31	20	28	7	15
0	11	16	18	38	36	51	36	75
Total	71	100	48	100	71	100	48*	100

* One missing.

**Table 3:** Number of different diagnoses and number of prescriptions recorded at consultations for patients in Öregrund-Östhammar 1996–1997

Number of diagnoses per subject	FGID (*n* = 176)	%	SSF (*n* = 71)	%	Number of prescriptions per subject	FGID (*n* = 385)	%	SSF (*n* = 167)	%
6–8	6	8	1	2	13–33	12	17	5	10
3–5	22	31	10	21	5–12	16	22	6	13
1–2	31	44	19	40	1–4	26	37	16	33
0	12	17	18	37	0	17	24	21	44
Total	71	100	48	100	Total	71	100	48	100

FGID, functional gastrointestinal disorder; SSF, strictly symptom free.

### Comorbidity

**Figure 1:**
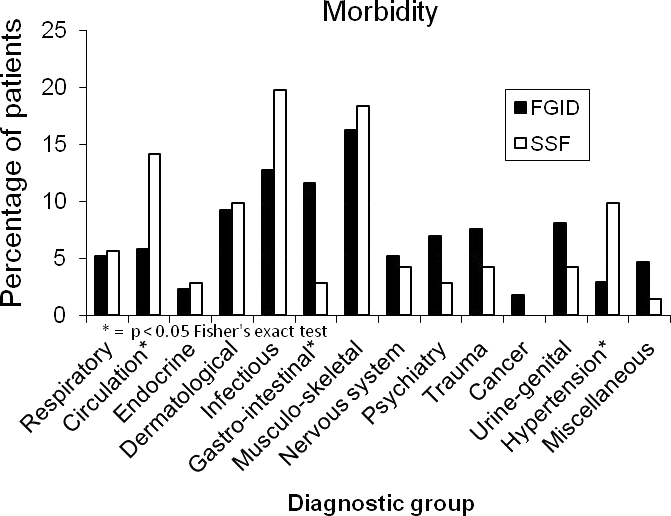
Morbidity expressed as the proportion of subjects (crude rate) with functional gastrointestinal disorder (FGID) and strictly symptom free (SSF) that had consulted for any ICD-9 diagnosis during 1996–1997. Univariate significance *p < 0.05, Fisher's exact test

The FGID patients had a non-GI diagnosis code recorded in a majority, 97%, of the consultations, compared with a non-GI diagnosis code in all consultations among SSF patients. Two SSF patients within the SSF had an additional GI diagnosis set: one with gastroesophageal reflux and one with meteorism. Comorbidity was presented as the number of different ICD-9 diagnostic groups in each consultation, for 410 consultations (FGID: *n* = 300; SSF: *n* = 110). There was no statistical significance in the distribution of set diagnoses for the 14 diagnostic groups determined by univariate comparison between the FGID and SSF groups, except for the GI, circulatory and hypertension diagnostic groups. In the latter two diagnostic groups, FGID patients had a lower mean age than SSF [circulation, 58 years (FGID) and 66 years (SSF), p = 0.049: hypertension, 63 years (FGID) and 75 years (SSF), p = 0.004]. The morbidity is illustrated in Figure 1.

### Referrals

There were 34 referrals recorded for the FGID and SSF groups, and none were referred more than twice. In the FGID group, 27% were referred to a specialist, compared with 21% in the SSF group (ns).

### Sick leave

There were no differences in either the number of sick leave episodes or the number of sick leave days for those with FGID and SSF, as highlighted in Table 2. The reason for sick leave for GI disorders was 9% for the FGID (of 956 days) and 0% for SSF (of 329 days) and thus the corresponding figures for non-GI disorders were 91% for the FGID and 100% for SSF.

### Anxiety and depression

Functional gastrointestinal disorders had increased levels of anxiety (p < 0.0001) and depression (p = 0.0001) and a significant age- and sex-adjusted higher risk for anxiety OR = 11.5 (CI: 1.4–96; p = 0.025) and for depression OR = 5.2 (CI: 1.1–25; 0.038) (see Table 1).

### Comparison with ROME II definition

The definition of dyspepsia was more restricted in terms of combinations of symptoms than the ROME II definition, but wider in terms of abdominal location, as not only epigastric but also midabdominal and flank symptoms were included. The IBS definition requires, aside from compulsory ‘abdominal pain and discomfort’ a combination of bowel habit disturbances (diarrhoea and/or constipation) and a symptom mainly labelled as ‘supportive’ in the ROME II definition of IBS).

Adopting the ROME II definitions for FGID (dyspepsia and IBS) as closely as possible, 4.8% of the 911 subjects in the population sample (18) were erroneously classified as having FGID instead of ‘minor symptoms’, with an overall agreement of 95.2% between the combinations of FGID definitions.

## Discussion

This study demonstrated that persistent FGID in the general population was related to increased comorbidity and increased healthcare consumption because of non-GI disorders. Subjects with FGID had more diagnoses, consultations and phone calls to their doctors and additional medication prescribed in primary and outpatient care than strictly GI symptom-free subjects did. The increased burden to healthcare was thus not explained by GI problems, but by the whole spectrum of diagnoses within the ICD-9 diagnostic groups also including the hypertensive and circulatory diseases as the difference found probably is a result of the older age of the SSF group. As it is not likely that FGID can be a cause of all different diseases, it seems that FGID in some way is related to somatic and psychological distress. Personality may also play an important part.

One strength of this study was the population-based approach, thus, avoiding sample bias because of healthcare-seeking behaviour in subjects with GI complaints (25). The sampling method with repeated reporting of dyspepsia or IBS twice for the FGID group (1995 and 1996), and up to four times for the SSF group (1988, 1989, 1995 and 1996), with the same type of questionnaire (ASQ), assured defined study groups and a precise measure of the outcome variables. The electronic medical records and the validated HADS questionnaire secured valid measurements of the exposure variables.

Aside from the slightly higher education level in the eastern part, there were no major differences between the area investigated and the remainder of Östhammar. As the population in the entire Östhammar region had a slightly lower educational level than the Swedish population (26), the sample from the eastern area was probably more representative of the general Swedish population. People in the eastern area made most outpatient and almost all non-specialist consultations within their own community area (data on file). Thus, the consultations made outside the catchment area could not bias the results.

The ASQ has been thoroughly validated and found reliable (4,27).The definitions of dyspepsia and IBS used in this study were those used in the original study from 1988 (28) when the first ROME criteria (13) were unavailable. The original study definitions were retained despite changes in later definitions, as it allowed comparison of the prevalence of diseases over time. The definitions used for FGID (dyspepsia and IBS) is in good concordance with the now used The Rome Foundation Criteria for the Functional GI Disorders (ROME) II classification as only 4.8% were erroneously classified as having FGID, with an overall agreement of 95.2% between the combinations of FGID definitions. Thus, the potential error of classification was considered insignificant for the conclusions.

This study was not a case–control study, but rather a study of all subjects with FGID compared with those repeatedly SSF within the population sample. Subjects with FGID were on average younger than SSF (18), as the prevalence of dyspepsia and IBS is higher in younger age groups (8): age was adjusted in the multivariate analysis and the mean age analysis. A power calculation was performed before the study but the study was not powered to detect differences in comorbidity which means a possibility that a type II error has led to a failure to find the statistically significant difference between some of the variables.

Healthcare-seeking behaviour is complex and its interaction with sick leave has been mainly studied in patient samples. Patients with non-ulcer dyspepsia are found to take excessive sick leave compared with ulcer patients (19), but only 23% of absenteeism was caused by GI complaints, compared with 9% in this study. Moreover, IBS patients with high comorbidity visit their GP more frequently than those with less comorbidity (29). Although the issue has been highlighted (17), it is mainly for IBS and is not population based.

We conclude that FGID is related to an increased demand on primary healthcare because of an increased overall comorbidity. Our findings indicate that FGID is a type of intestinal reaction, related to somatic and psychological distress in a subgroup of subjects. To the authors’ knowledge there is no prior study comparing persistently symptomatic FGID with long-lasting symptom-free subjects. The results could be generalised to the complete Swedish population, as the study groups sampled were from a well-defined and thoroughly investigated population.

To re-assure the patient and avoid unnecessary and expensive investigations, the treatment of people with FGID should be through a holistic healthcare approach. Specialist care focusing solely on GI problems may miss the target.

## Supplementary material

The following supplementary material is available for this article:

**Slide Show S1.** A short slide presentation of the article.

This material is available as part of the online article from: http://www.blackwell-synergy.com/

## References

[1] Jones R, Lydeard SE, Hobbs FD (1990). Dyspepsia in England and Scotland. Gut.

[2] Talley NJ, Zinsmeister AR, Schleck CD, Melton LJd (1992). Dyspepsia and dyspepsia subgroups: a population-based study. Gastroenterology.

[3] Agréus L (1993). Socio-economic factors, health care consumption and rating of abdominal symptom severity. A report from the Abdominal Symptom Study. Fam Pract.

[4] Agréus L, Svärdsudd K, Nyrén O, Tibblin G (1995). Irritable bowel syndrome and dyspepsia in the general population: overlap and lack of stability over time. Gastroenterology.

[5] Levy RL, Von Korff M, Whitehead WE (2001). Costs of care for irritable bowel syndrome patients in a health maintenance organization. Am J Gastroenterol.

[6] Agreus L, Borgquist L (2002). The cost of gastro-oesophageal reflux disease, dyspepsia and peptic ulcer disease in Sweden. Pharmacoeconomics.

[7] McDougall NI, Johnston BT, Kee F, Collins JS, McFarland RJ, Love AH (1996). Natural history of reflux oesophagitis: a 10 year follow up of its effect on patient symptomatology and quality of life. Gut.

[8] Agreus L, Svardsudd K, Talley NJ, Jones MP, Tibblin G (2001). Natural history of gastroesophageal reflux disease and functional abdominal disorders: a population-based study. Am J Gastroenterol.

[9] van Bommel MJ, Numans ME, de Wit NJ, Stalman WA (2001). Consultations and referrals for dyspepsia in general practice – a one year database survey. Postgrad Med J.

[10] Agréus L (2002). Natural history of dyspepsia. Gut.

[11] Drossman DA, Corazziari E, Thompson WG, Talley NJ, Whitehead W (2000). ROME II. The Functional Gastrointestinal Disorders.

[12] Aro P, Storskrubb T, Ronkainen J (2006). Peptic ulcer disease in a general adult population. The Kalixanda study: a random population-based study. Am J Epidemiol.

[13] Talley N, Stanghellini V, Heading R, Koch K, Malagelada J, Tytgat G (1999). Functional gastroduodenal disorders: a working team report for the ROME II consensus on functional gastrointestinal disorders. Gut.

[14] Holtmann G, Goebell H, Talley NJ (1997). Functional dyspepsia and irritable bowel syndrome: is there a common pathophysiological basis?. Am J Gastroenterol.

[15] Whitehead WE, Gibbs NA, Li Z, Drossman DA (1998). Is functional dyspepsia just a subset of the irritable bowel syndrome?. Baillieres Clin Gastroenterol.

[16] Koloski NA, Talley NJ, Boyce PM (2002). Epidemiology and health care seeking in the functional GI disorders: a population-based study. Am J Gastroenterol.

[17] Whitehead WE, Palsson O, Jones KR (2002). Systematic review of the comorbidity of irritable bowel syndrome with other disorders: what are the causes and implications?. Gastroenterology.

[18] Alander T, Svardsudd K, Johansson SE, Agreus L (2005). Psychological illness is commonly associated with functional gastrointestinal disorders and is important to consider during patient consultation: a population-based study. BMC Med.

[19] Nyrén O, Adami HO, Gustavsson S, Lööf L (1986). Excess sick-listing in nonulcer dyspepsia. J Clin Gastroenterol.

[20] Agréus L (1993). The Abdominal Symptom Study. An Epidemiological Survey of Gastrointestinal and Other Abdominal Symptoms in the Adult Population of Östhammar, Sweden.

[21] Talley NJ, Colin-Jones D, Koch KJ, Koch M, Nyrén O, Stanghellini V (1991). Functional dyspepsia: a classification with guidelines for diagnoses and management. MAIN ROME. Gastroenterol Int.

[22] Zigmond AS, Snaith RP (1983). The Hospital Anxiety and Depression Scale. Acta Psychiatr Scand.

[23] Herrmann C (1997). International experiences with the Hospital Anxiety and Depression Scale – a review of validation data and clinical results. J Psychosom Res.

[24] Stata Corporation (2002). Stata Statistical Software. Release 8.0.

[25] Lydeard S, Jones R (1989). Factors affecting the decision to consult with dyspepsia: comparison of consulters and non-consulters. J R Coll Gen Pract.

[26] Statistiska centralbyrån (SCB) (2004). Utbildningsregistret, 2004, 25–64 år.

[27] Agreus L, Svardsudd K, Nyren O, Tibblin G (1993). Reproducibility and validity of a postal questionnaire. The abdominal symptom study. Scand J Prim Health Care.

[28] Agréus L, Nyrén O, Svärdsudd K, Tibblin G (1990). Ont i magen-En epidemiologisk studie om bukbesvär i Östhammars kommun. Sven Läkarsällskapets Hygea.

[29] Vandvik PO, Wilhelmsen I, Ihlebaek C, Farup PG (2004). Comorbidity of irritable bowel syndrome in general practice: a striking feature with clinical implications. Aliment Pharmacol Ther.

