# Supplementary material for: Functional gastrointestinal disorder is associated with increased non-gastrointestinal healthcare consumption in the general population
Source: Int J Clin Pract. 2008 Feb;62(2):234–40. doi: 10.1111/j.1742-1241.2007.01549.x (PMC2657998; doi:10.1111/j.1742-1241.2007.01549.x)
Supplement: Supplementary file 1 [file ijcp0062-0234-SD1.ppt]

## Slide 1
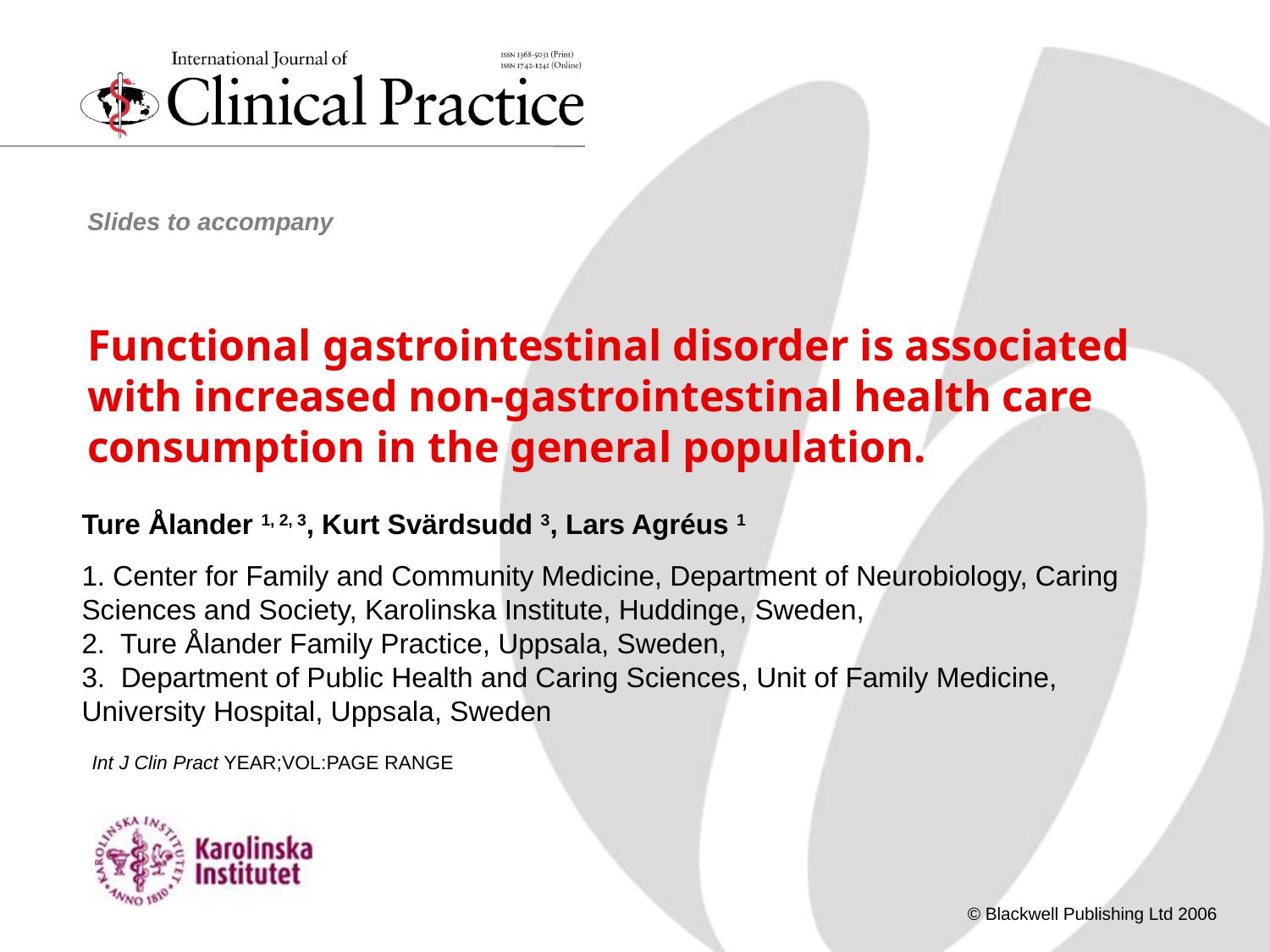

# Functional gastrointestinal disorder is associated with increased non-gastrointestinal health care consumption in the general population.
Ture Ålander 1, 2, 3, Kurt Svärdsudd 3, Lars Agréus 1
1. Center for Family and Community Medicine, Department of Neurobiology, Caring Sciences and Society, Karolinska Institute, Huddinge, Sweden,
2. Ture Ålander Family Practice, Uppsala, Sweden,
3. Department of Public Health and Caring Sciences, Unit of Family Medicine, University Hospital, Uppsala, Sweden
Int J Clin Pract YEAR;VOL:PAGE RANGE

## Slide 2
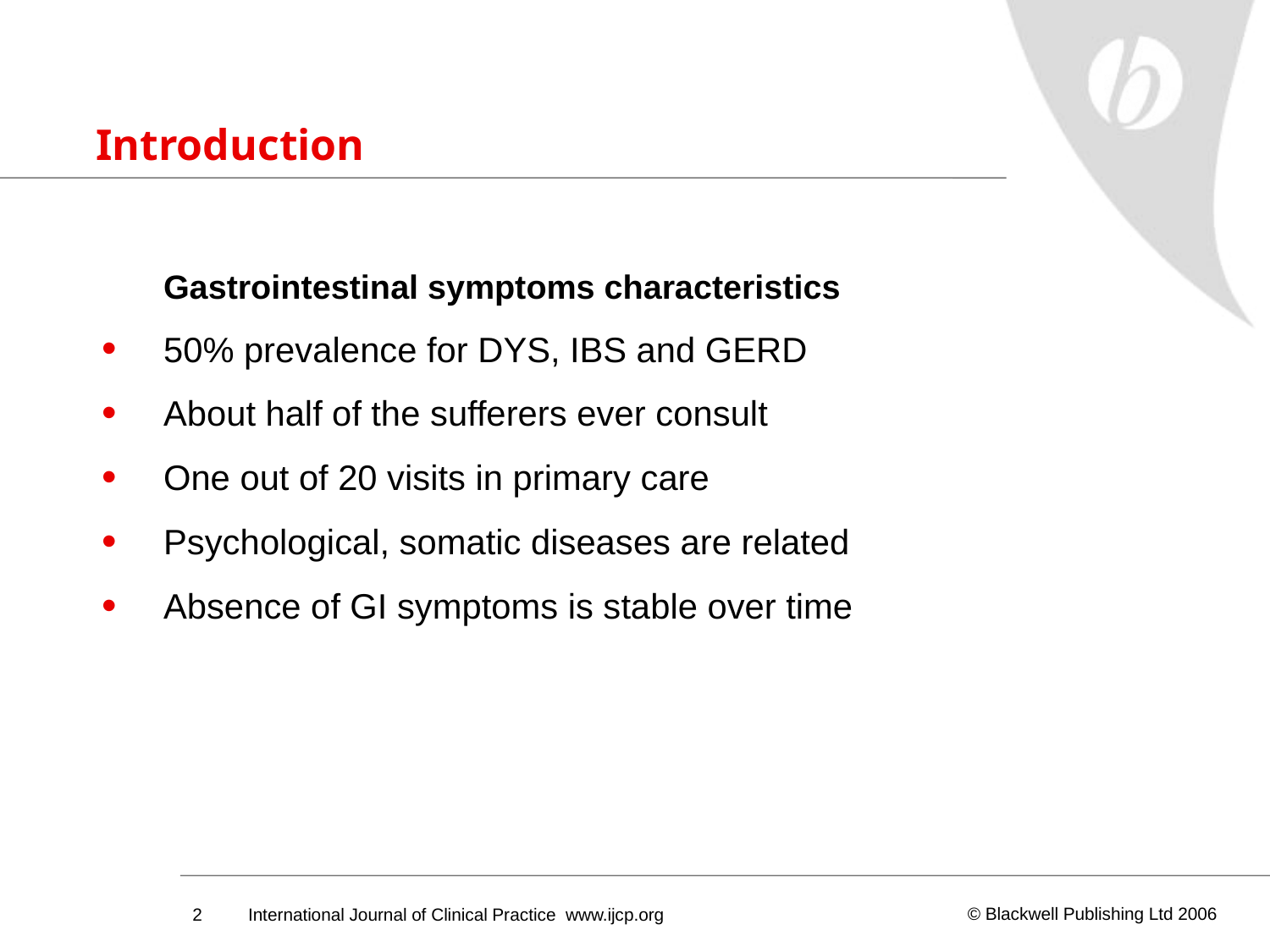

# Introduction
Gastrointestinal symptoms characteristics
50% prevalence for DYS, IBS and GERD
About half of the sufferers ever consult
One out of 20 visits in primary care
Psychological, somatic diseases are related
Absence of GI symptoms is stable over time
International Journal of Clinical Practice www.ijcp.org
<number>

## Slide 3
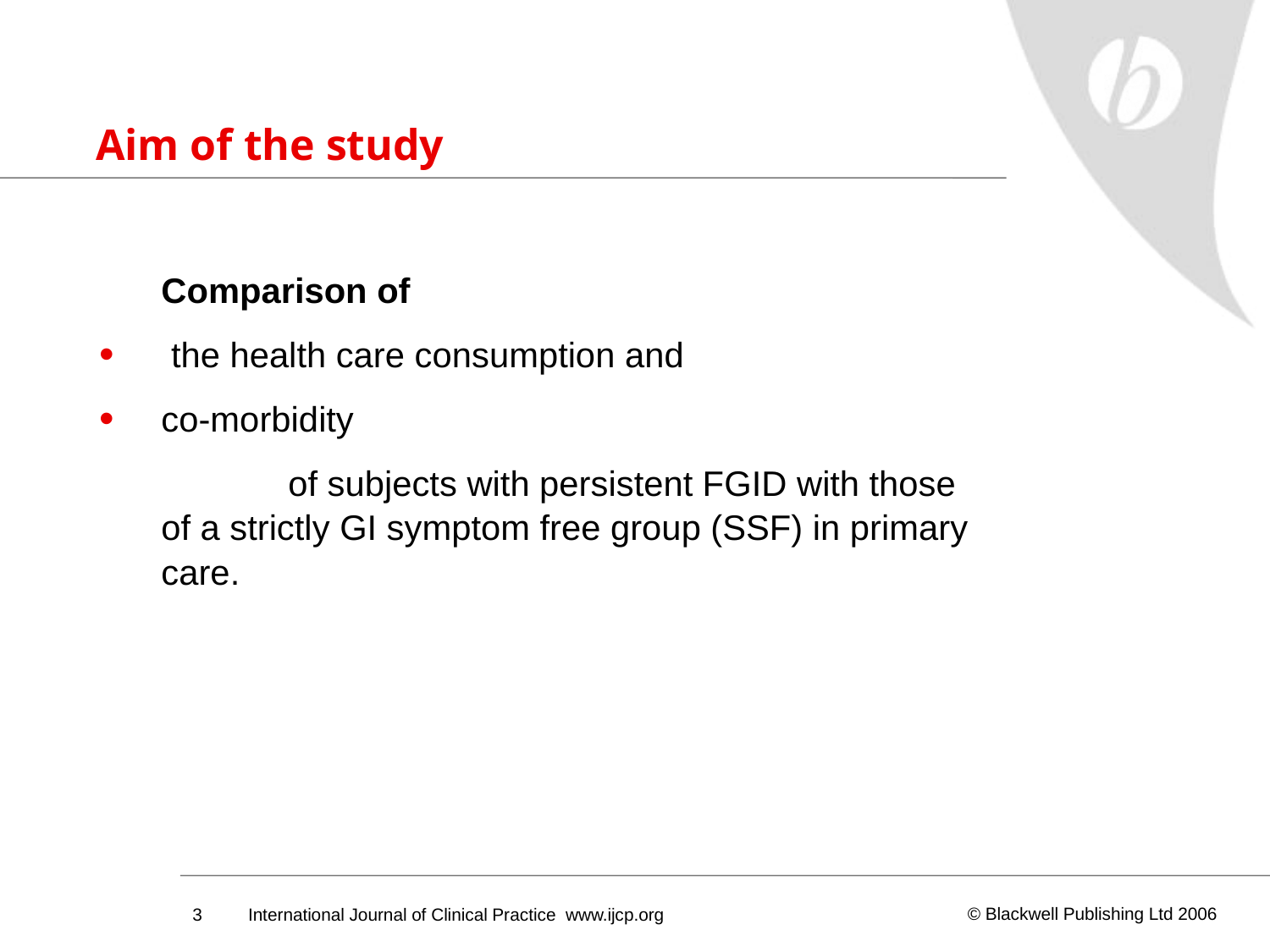

# Aim of the study
Comparison of
 the health care consumption and
co-morbidity
	of subjects with persistent FGID with those of a strictly GI symptom free group (SSF) in primary care.
International Journal of Clinical Practice www.ijcp.org
<number>

## Slide 4
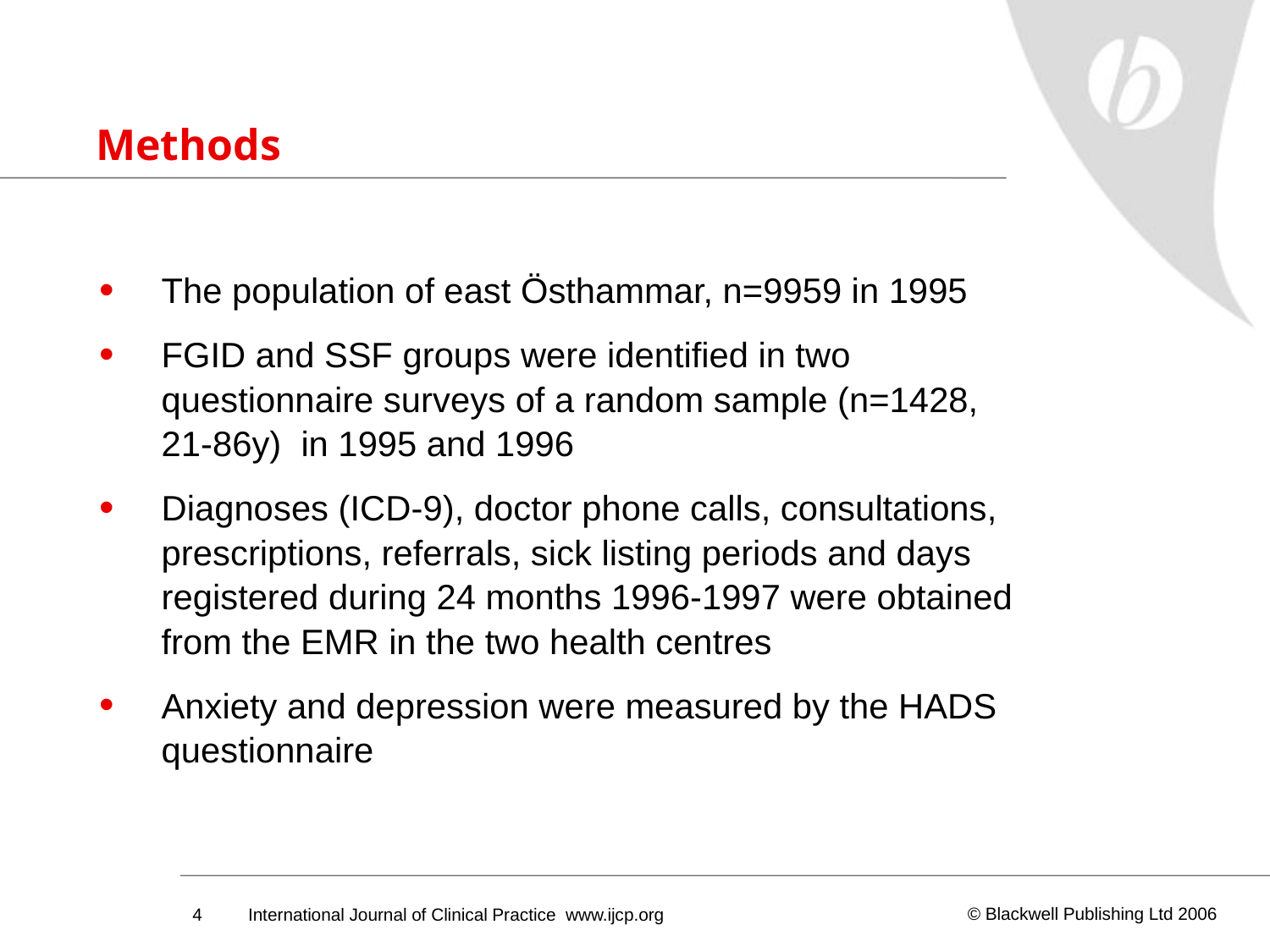

# Methods
The population of east Östhammar, n=9959 in 1995
FGID and SSF groups were identified in two questionnaire surveys of a random sample (n=1428, 21-86y) in 1995 and 1996
Diagnoses (ICD-9), doctor phone calls, consultations, prescriptions, referrals, sick listing periods and days registered during 24 months 1996-1997 were obtained from the EMR in the two health centres
Anxiety and depression were measured by the HADS questionnaire
International Journal of Clinical Practice www.ijcp.org
<number>

## Slide 5
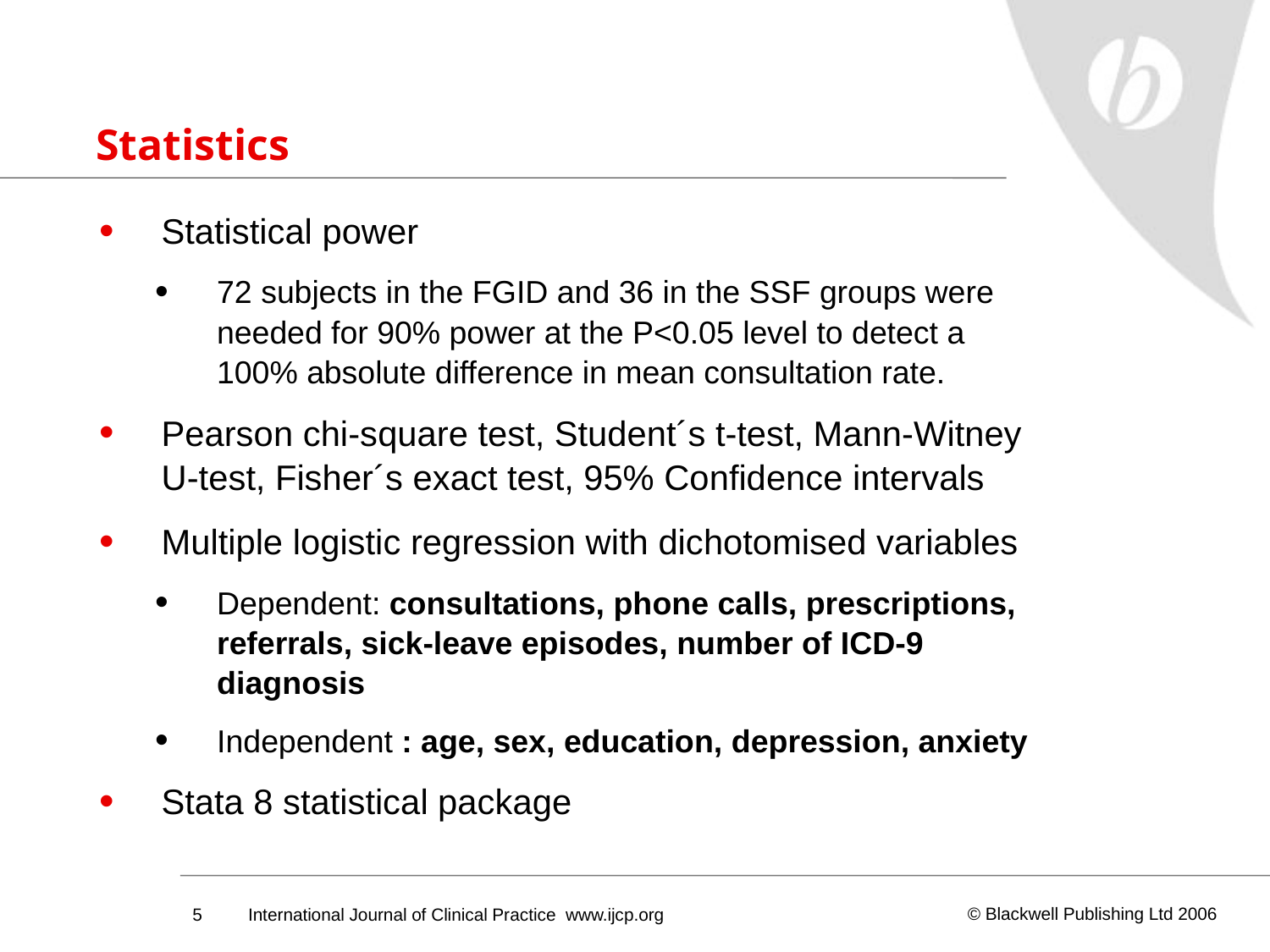

# Statistics
Statistical power
72 subjects in the FGID and 36 in the SSF groups were needed for 90% power at the P<0.05 level to detect a 100% absolute difference in mean consultation rate.
Pearson chi-square test, Student´s t-test, Mann-Witney U-test, Fisher´s exact test, 95% Confidence intervals
Multiple logistic regression with dichotomised variables
Dependent: consultations, phone calls, prescriptions, referrals, sick-leave episodes, number of ICD-9 diagnosis
Independent : age, sex, education, depression, anxiety
Stata 8 statistical package
International Journal of Clinical Practice www.ijcp.org
<number>

## Slide 6
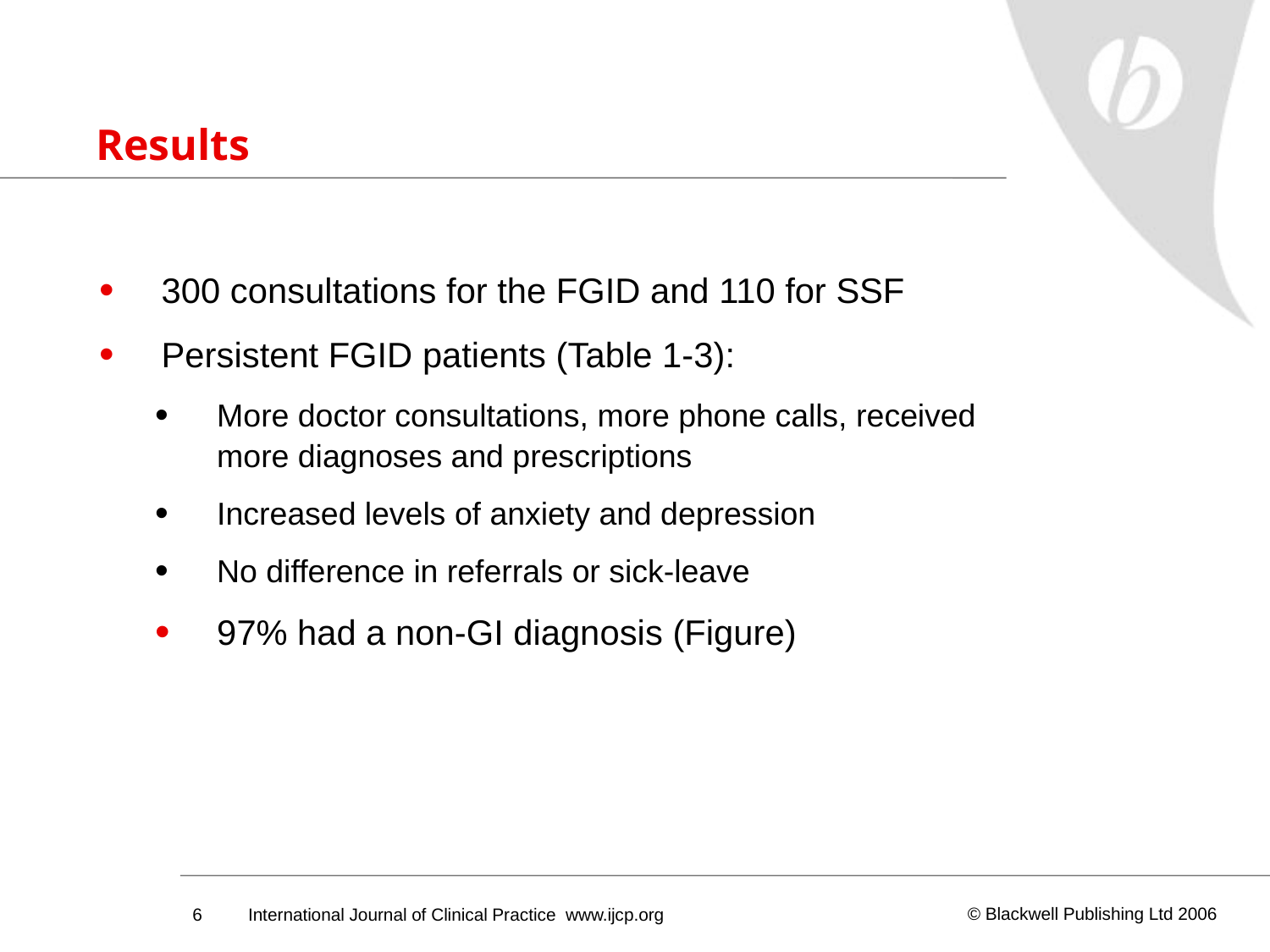

# Results
300 consultations for the FGID and 110 for SSF
Persistent FGID patients (Table 1-3):
More doctor consultations, more phone calls, received more diagnoses and prescriptions
Increased levels of anxiety and depression
No difference in referrals or sick-leave
97% had a non-GI diagnosis (Figure)
International Journal of Clinical Practice www.ijcp.org
<number>

## Slide 7
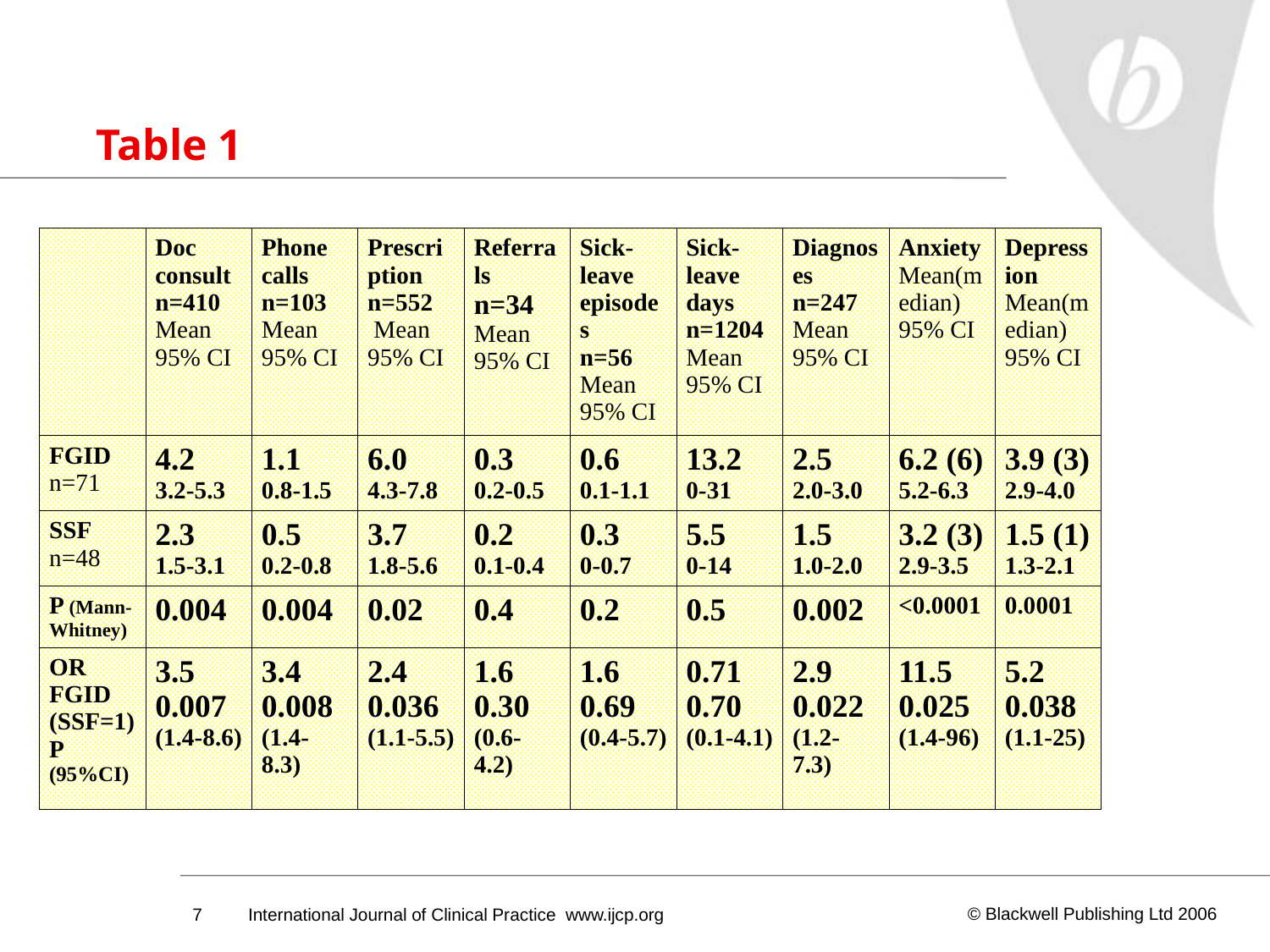

# Table 1
| | Doc consult n=410 Mean 95% CI | Phone calls n=103 Mean 95% CI | Prescription n=552 Mean 95% CI | Referrals n=34 Mean 95% CI | Sick-leave episodes n=56 Mean 95% CI | Sick-leave days n=1204 Mean 95% CI | Diagnoses n=247 Mean 95% CI | Anxiety Mean(median) 95% CI | Depression Mean(median) 95% CI |
| --- | --- | --- | --- | --- | --- | --- | --- | --- | --- |
| FGID n=71 | 4.2 3.2-5.3 | 1.1 0.8-1.5 | 6.0 4.3-7.8 | 0.3 0.2-0.5 | 0.6 0.1-1.1 | 13.2 0-31 | 2.5 2.0-3.0 | 6.2 (6) 5.2-6.3 | 3.9 (3) 2.9-4.0 |
| SSF n=48 | 2.3 1.5-3.1 | 0.5 0.2-0.8 | 3.7 1.8-5.6 | 0.2 0.1-0.4 | 0.3 0-0.7 | 5.5 0-14 | 1.5 1.0-2.0 | 3.2 (3) 2.9-3.5 | 1.5 (1) 1.3-2.1 |
| P (Mann-Whitney) | 0.004 | 0.004 | 0.02 | 0.4 | 0.2 | 0.5 | 0.002 | <0.0001 | 0.0001 |
| OR FGID (SSF=1) P (95%CI) | 3.5 0.007 (1.4-8.6) | 3.4 0.008 (1.4-8.3) | 2.4 0.036 (1.1-5.5) | 1.6 0.30 (0.6-4.2) | 1.6 0.69 (0.4-5.7) | 0.71 0.70 (0.1-4.1) | 2.9 0.022 (1.2-7.3) | 11.5 0.025 (1.4-96) | 5.2 0.038 (1.1-25) |
International Journal of Clinical Practice www.ijcp.org
<number>

## Slide 8
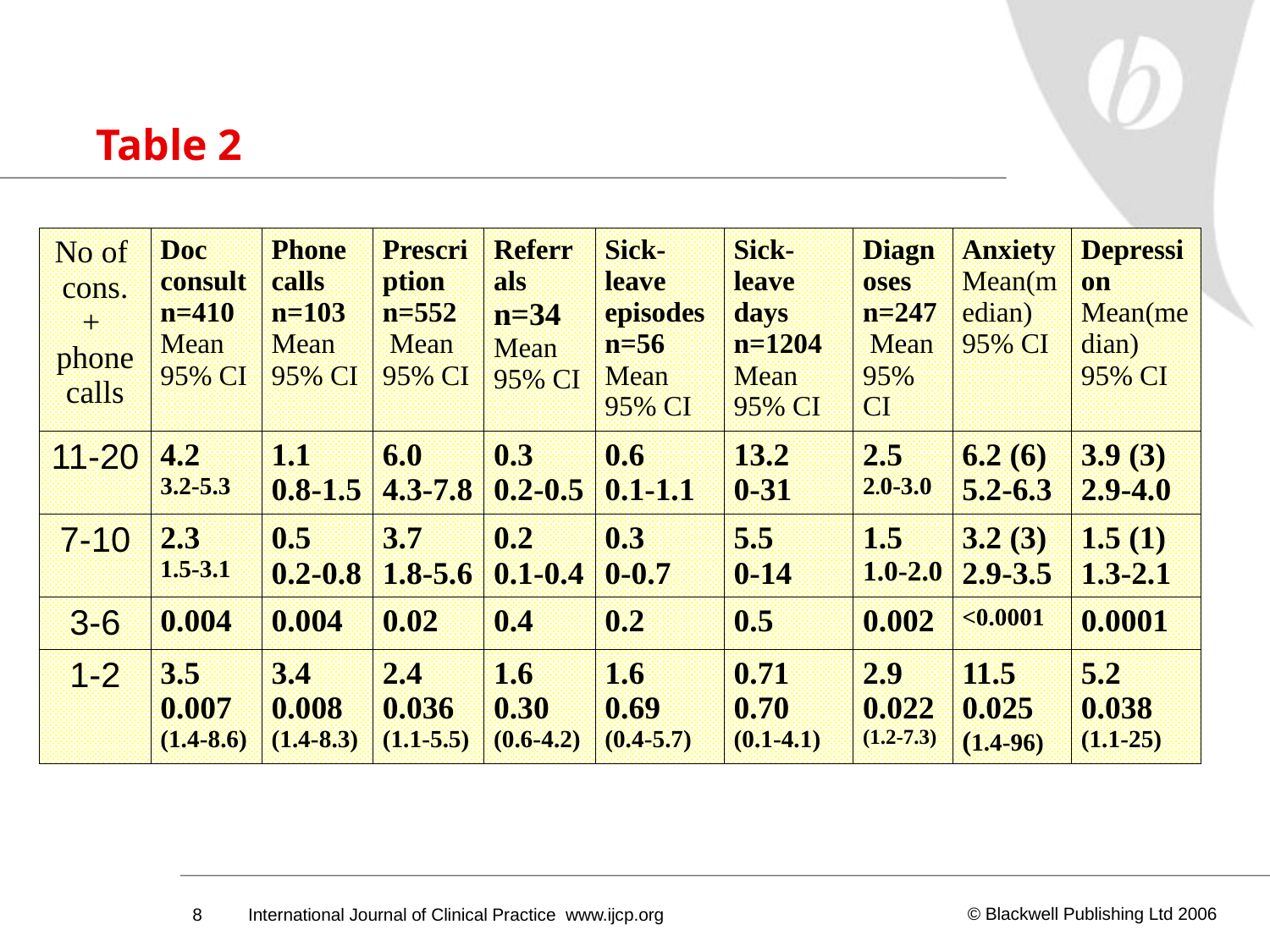

# Table 2
| No of cons. + phone calls | Doc consult n=410 Mean 95% CI | Phone calls n=103 Mean 95% CI | Prescription n=552 Mean 95% CI | Referrals n=34 Mean 95% CI | Sick-leave episodes n=56 Mean 95% CI | Sick-leave days n=1204 Mean 95% CI | Diagnoses n=247 Mean 95% CI | Anxiety Mean(median) 95% CI | Depression Mean(median) 95% CI |
| --- | --- | --- | --- | --- | --- | --- | --- | --- | --- |
| 11-20 | 4.2 3.2-5.3 | 1.1 0.8-1.5 | 6.0 4.3-7.8 | 0.3 0.2-0.5 | 0.6 0.1-1.1 | 13.2 0-31 | 2.5 2.0-3.0 | 6.2 (6) 5.2-6.3 | 3.9 (3) 2.9-4.0 |
| 7-10 | 2.3 1.5-3.1 | 0.5 0.2-0.8 | 3.7 1.8-5.6 | 0.2 0.1-0.4 | 0.3 0-0.7 | 5.5 0-14 | 1.5 1.0-2.0 | 3.2 (3) 2.9-3.5 | 1.5 (1) 1.3-2.1 |
| 3-6 | 0.004 | 0.004 | 0.02 | 0.4 | 0.2 | 0.5 | 0.002 | <0.0001 | 0.0001 |
| 1-2 | 3.5 0.007 (1.4-8.6) | 3.4 0.008 (1.4-8.3) | 2.4 0.036 (1.1-5.5) | 1.6 0.30 (0.6-4.2) | 1.6 0.69 (0.4-5.7) | 0.71 0.70 (0.1-4.1) | 2.9 0.022 (1.2-7.3) | 11.5 0.025 (1.4-96) | 5.2 0.038 (1.1-25) |
International Journal of Clinical Practice www.ijcp.org
<number>

## Slide 9
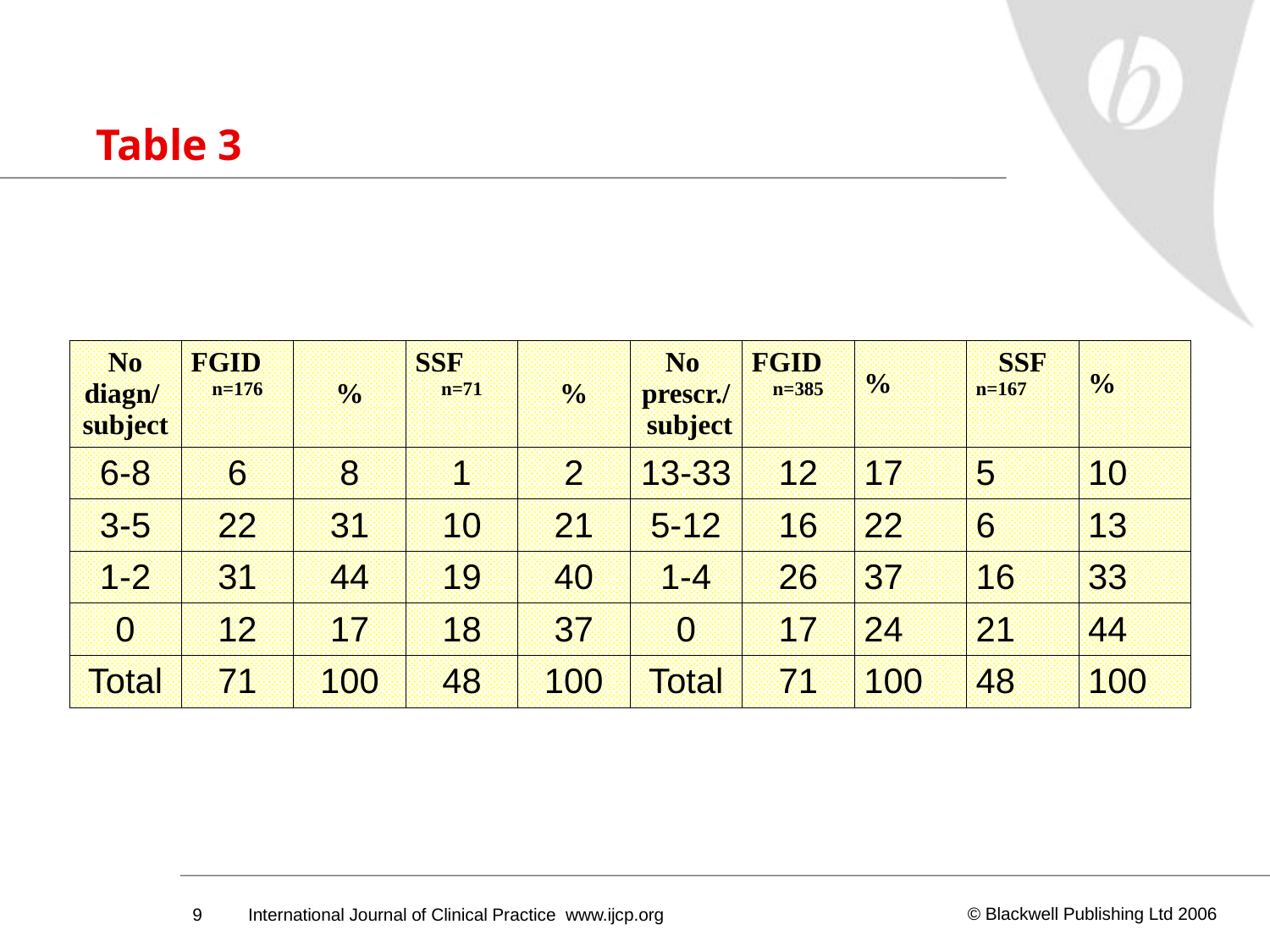

# Table 3
| No diagn/ subject | FGID n=176 | % | SSF n=71 | % | No prescr./ subject | FGID n=385 | % | SSF n=167 | % |
| --- | --- | --- | --- | --- | --- | --- | --- | --- | --- |
| 6-8 | 6 | 8 | 1 | 2 | 13-33 | 12 | 17 | 5 | 10 |
| 3-5 | 22 | 31 | 10 | 21 | 5-12 | 16 | 22 | 6 | 13 |
| 1-2 | 31 | 44 | 19 | 40 | 1-4 | 26 | 37 | 16 | 33 |
| 0 | 12 | 17 | 18 | 37 | 0 | 17 | 24 | 21 | 44 |
| Total | 71 | 100 | 48 | 100 | Total | 71 | 100 | 48 | 100 |
International Journal of Clinical Practice www.ijcp.org
<number>

## Slide 10
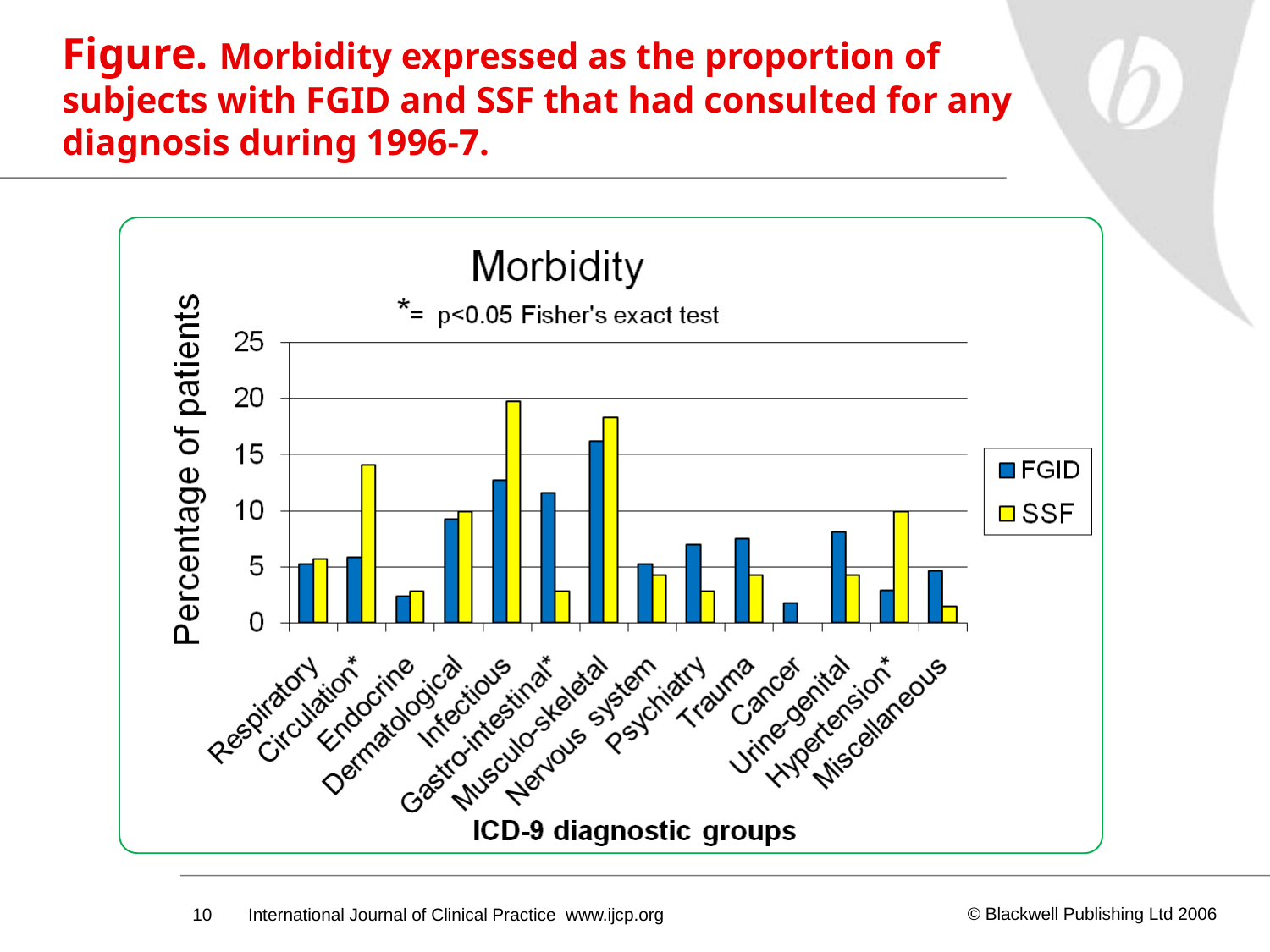

# Figure. Morbidity expressed as the proportion of subjects with FGID and SSF that had consulted for any diagnosis during 1996-7.
International Journal of Clinical Practice www.ijcp.org
<number>

## Slide 11
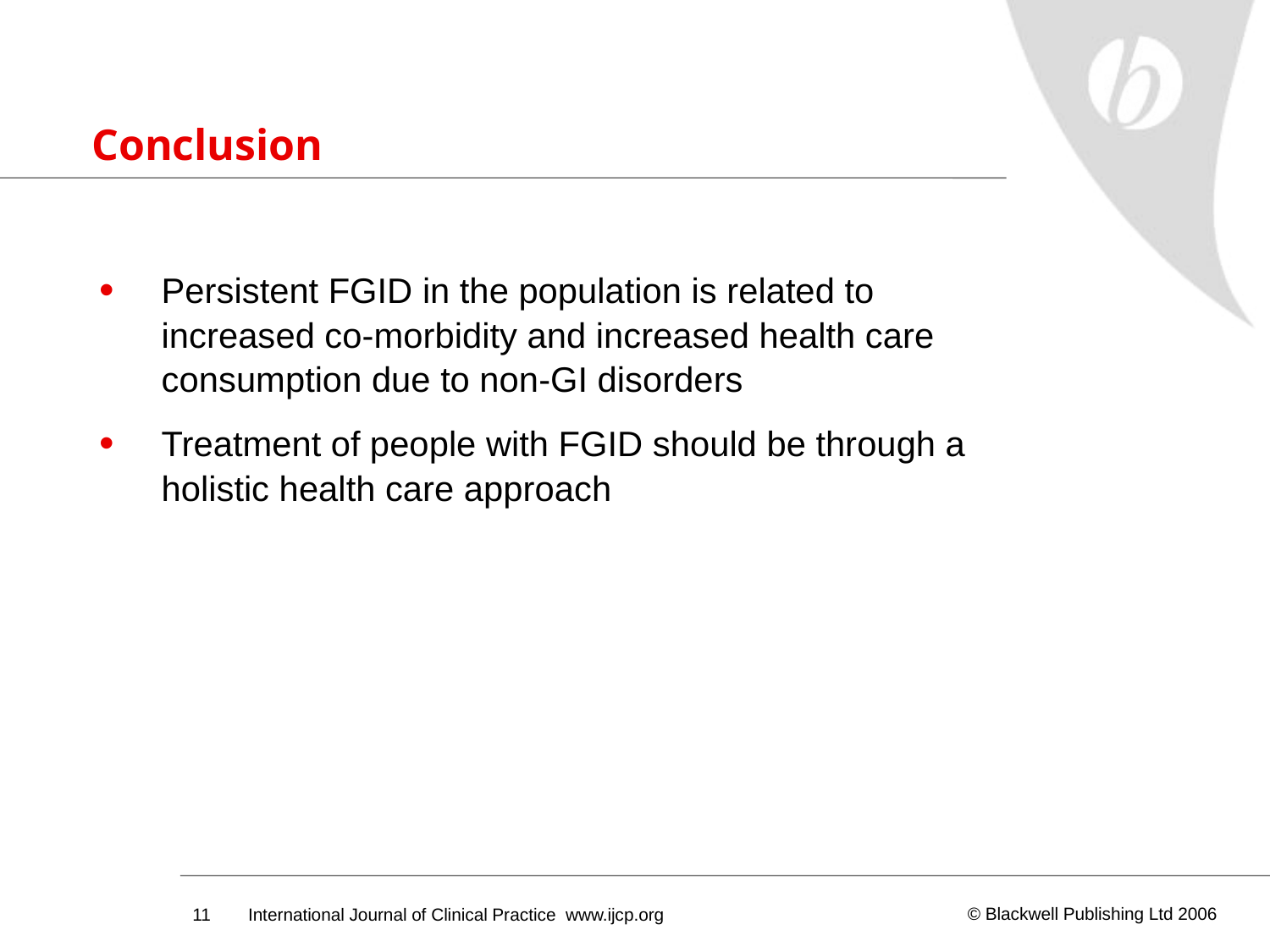

# Conclusion
Persistent FGID in the population is related to increased co-morbidity and increased health care consumption due to non-GI disorders
Treatment of people with FGID should be through a holistic health care approach
International Journal of Clinical Practice www.ijcp.org
<number>
